# Three-Dimensional Gait Analysis Can Shed New Light on Walking in Patients with Haemophilia

**DOI:** 10.1155/2013/284358

**Published:** 2013-05-13

**Authors:** Sébastien Lobet, Christine Detrembleur, Firas Massaad, Cedric Hermans

**Affiliations:** ^1^Haemostasis and Thrombosis Unit, Saint-Luc University Clinics, 1200 Brussels, Belgium; ^2^Physical Medicine and Rehabilitation Unit, Saint-Luc University Clinics, 1200 Brussels, Belgium; ^3^Institute of Neuroscience (IoNS), Catholic University of Louvain, 1200 Brussels, Belgium; ^4^Department of Biomedical Kinesiology, Faculty of Kinesiology and Rehabilitation Sciences, KU Leuven, 3001 Heverlee, Belgium

## Abstract

In patients with haemophilia (PWH) (from Greek “blood love”), the long-term consequences of repeated haemarthrosis include cartilage damage and irreversible arthropathy, resulting in severe impairments in locomotion. Quantifying the extent of joint damage is therefore important in order to prevent disease progression and compare the efficacy of treatment strategies. Musculoskeletal impairments in PWH may stem from structural and functional abnormalities, which have traditionally been evaluated radiologically or clinically. However, these examinations are performed in a supine position (i.e., non-weight-bearing condition). We therefore suggest three-dimensional gait analysis (3DGA) as an innovative approach designed to focus on the functional component of the joint during the act of walking. This is of the utmost importance, as pain induced by weight-bearing activities influences the functional performance of the arthropathic joints significantly. This review endeavors to improve our knowledge of the biomechanical consequences of multiple arthropathies on gait pattern in adult patients with haemophilia using 3DGA. In PWH with arthropathy, the more the joint function was altered, the more the metabolic energy was consumed. 3DGA analysis could highlight the effect of an orthopedic disorder in PWH during walking. Indeed, mechanical and metabolic impairments were correlated to the progressive loss of active mobility into the joints.

## 1. Introduction

Haemophilia (from the Greek haima “blood” and philia “love”) is a group of hereditary genetic disorders that impair the body's ability to control blood clotting or coagulation. However, ironically, if you were to ask any patient with haemophilia (PWH) how they feel about the disorder, they would probably reply that their feelings towards blood are anything but love.

Although brain haemorrhage and bleeding into internal organs represent major threats to the life of PWH, approximately 80%–90% of bleeding episodes occur in the musculoskeletal (MSK) system, especially in the large synovial joints, as well as in the muscles, thus constituting the principal health problem. This induces progressive cartilage damage, leading to joint destruction and subsequent severe functional limitation.

Treatment and, ideally, the prevention of MSK are the main challenges in PWH. The adequate prevention of MSK complications requires the early detection of the first signs of joint impairment in relatively asymptomatic patients as well as the efficient followup of MSK complications already present. Appropriate treatment, whether haemostatic or orthopedic, is only possible if we have reliable assessment tools at our disposal which can make it possible to quantify the benefits of such treatment. 

The MSK assessment has traditionally been evaluated using both radiological and clinical joint scoring systems [1–5]. Information obtained from these scores is regularly used in clinical practice to evaluate the effects of different treatments on the progression of arthropathy, including clotting factor prophylaxis, physical therapy, and surgical procedures [6]. However, clinical joint scoring may not be sensitive to subtle changes in joint status and radiological examination does not provide an understanding of the causes underlying the impairments.

Recently, an interest in the biomechanical status of hemophilic joints has emerged. In this review, we explored a new approach to the functional assessment of MSK complications in PWH by means of a specialized laboratory equipment to assess human motor performance with a three-dimensional gait analysis (3DGA). Gait is the pattern of movement of the limbs of animals, including humans, during locomotion. Due to the rapidity of movement, simple direct observation is rarely sufficient to give any insight into the pattern of limb movement or to determine the biomechanical causes of an abnormal gait in humans (biomechanics being the study of the structure and function of biological systems). In 3DGA, physics and mathematics are applied to unravel the biomechanics of a pathological human gait and pinpoint which joint or muscle system is responsible for the functional deficit. Contrary to radiological and clinical examinations performed in a supine position, the uniqueness of 3DGA is that it assesses the patient during the act of walking, that is, under weight-bearing conditions. This is of the utmost importance, as pain induced by weight-bearing activities significantly influences the functional performance of the arthropathic joints.

## 2. Method

**Figure 1:**
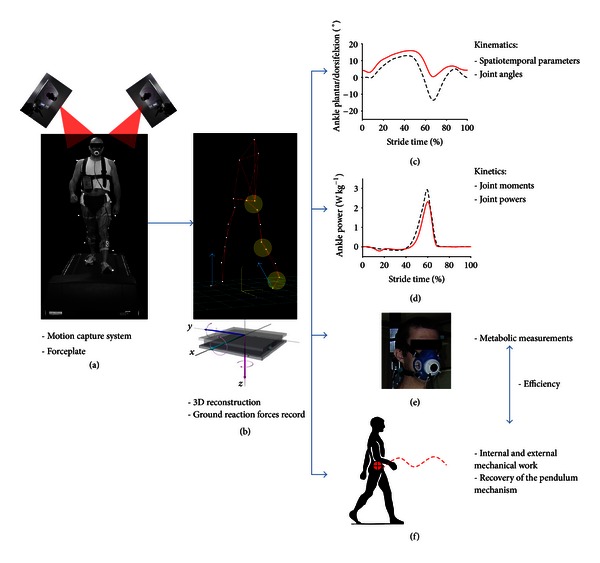
The infrared cameras (a) are positioned so that at least two visualize each reflective marker at any given time. From the reflective markers movements we can calculate the 3D trajectories of the body segments (b). The images are then processed to derive the graphs of the kinematics, that is, the joint range of movement of each lower limb joint (c). A force platform located under the treadmill (b) records the patient's ground reaction forces. The joint moments and powers, that is, kinetic data, (d) are derived from force platform measurements and kinematic data. Energy expenditure is measured indirectly based on the rate of oxygen consumption by the patient using an ergospirometer (e). Finally, the mechanical work is calculated as the work performed by muscles to raise and accelerate the center of body mass (external mechanical work) and to move the body segments relative to the center of body mass (internal mechanical work) (f).

To do so we ask the patient to walk on a treadmill equipped with force sensors that measure the ground reaction forces (GRF) under the patients feet while the patients are filmed by infrared cameras that track and record the trajectories of reflective markers positioned on the skin to define body segments (Figure 1(a)). This video-based motion analysis system measures the three-dimensional kinematics locomotion (kinematics being the branch of classical mechanics that describes the motion of points, bodies (objects), and systems of bodies (groups of objects) without consideration of the causes of motion). From the markers' position, it becomes possible to calculate the 3D trajectory of the marker in time and space which allows to calculate the joint angles and range of motion (ROM) (Figures 1(a) and 1(c)) between two adjacent segments in a particular anatomical plane (e.g., the ankle ROM is defined by the angle between the leg and foot segments in sagittal, frontal, or transversal planes).

Kinetics is the branch of physics that studies the motion of masses in relation to the forces acting on them. Taking direct measurements of muscle forces is not practical, as they require invasive procedures. From the GRF we could obtain kinetic values, such as the moments of force and power generated or absorbed at the major joint muscles (Figure 1(d)).

Kinematics and kinetics can assess the disease consequences at a single joint level (ankle, knee, or hip), without reflecting the impact on the body as a whole. The goals of achieving a normal gait are not only to decrease the stress on the muscles and joints but more importantly to decrease the energy required to move from place to place [7]. Indeed, most animals and humans use a variety of gaits to minimize energy consumption. In patients, gait abnormalities after neurologic or orthopedic disorders can make them consume two to three times more energy to walk in comparison to healthy individuals [8]. The gait laboratory at the Cliniques Universitaires Saint-Luc in Brussels specifically aims to evaluate the effects of joint impairments on global body function by calculating more “general” variables, such as mechanical work (net energy produced by muscles) and metabolic energy consumption (net chemical energy consumed by muscles). The total mechanical work is calculated as the work performed by muscles to raise and accelerate the center of body mass (external work) (CoM) and to move the body segments relative to the center of body mass (internal work) (Figure 1(f)) [9]. Energy expenditure is measured indirectly based on the rate of oxygen consumption (VO_2_) using an ergospirometer (Figure 1(e)). The net metabolic cost (i.e., the energy consumed by the muscles per unit of distance) is calculated as the net oxygen consumption over walking speed. The efficiency is calculated as the ratio between the total mechanical work and the net metabolic cost. A low efficiency means that a lot of energy is lost to produce a specific mechanical work by the muscles to walk. The goals of achieving a normal gait are not only to decrease the stress on the muscles and joints but more importantly to decrease the energy required to move [7].

**Figure 2:**
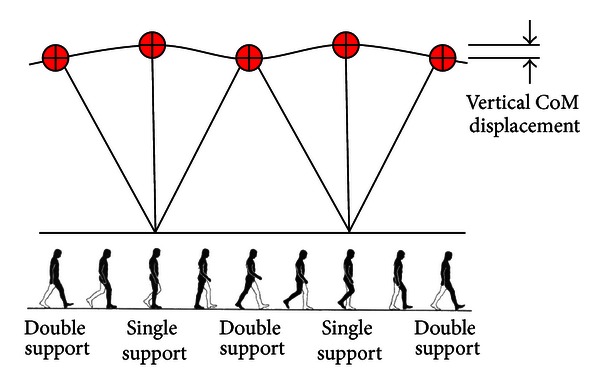
Inverted pendulum model of gait, showing how the center of body mass (CoM) rises during the single support and falls during the double support.

People walk in a style that consists in moving the whole body up and down which can easily be seen in the humans' head bobbing up and down while walking on the street for example. Why do we move vertically to walk straight ahead? In walking, the CoM (located approximately just above and between the hip joints) is lifted up and down during a walking stride (beginning and ending with the same foot contact with the ground). The pathway of the CoM is a smooth sinusoidal regular curve that moves up and down in the sagittal plane with an average rise and fall of about 3-4 cm (Figure 2) [10]. The CoM reaches its highest position when standing on one leg and its lowest position when both feet are in contact with the ground. This vertical movement of the CoM enables individuals to save energy, because we slow down as we rise and speed up as we fall, thus passively recovering kinetic energy as gravitational potential energy and back again, as in an inverted pendulum. The recovery index is indicative of the efficacy of this gait mechanism. At preferred walking speed, as much as 60%–65% of the required external mechanical energy may be recovered owing to this energy saving mechanism [11]. The other 35%–40% of external mechanical energy is lost from the system and must be supplied by the muscles. This pendulum-like mode of walking reduces the mechanical work actively supplied by the muscles to walking and, thus, it is supposed to save metabolic energy.

## 3. Results and Discussion

### 3.1. Psychometric Characteristics of 3DGA Tested in PWH

The correlations between structural alterations (determined by the Arnold-Hilgartner radiological score [1], the Pettersson radiological score [5], and the WFH-Gilbert clinical score [2]) and functional alterations (assessed by 3DGA and the Foot Function Index Revised short form, a validated self-reported ankle function assessment [12]) were explored in terms of PWH with ankle arthropathy [13]. Significant correlations were found between the self-reported functional assessment and three 3DGA variables representative of joint function, that is, ankle ROM in the sagittal plane, muscular moment, and power. These correlations proved that 3DGA appeared to measure what it really aimed to measure, namely, the function of joints.

In the context “body structure and function” domain of the International Classification of Functioning, Disability and Health (ICF) [14], we intended to clarify the relationship between the structural and functional assessments of a joint. Radiological and clinical scores were compared to ankle muscle peak power, considered as the most reliable 3DGA variable for ankle function. No significant relationships were found between clinical and functional scores on one hand and ankle power on the other hand, thereby confirming the absence of a direct link between the structural changes of a joint and its real functional potentiality [13]. The rehabilitation setting, in addition to clinical studies, continues to place much weight on the structural assessment of the joint when considering patient evolution and treatment efficacy. This observation supports the notion that the clinical practitioner should likewise focus on the functional aspects of the joint.

The natural progression of haemophilic arthropathy can also be evaluated using 3DGA. For this, adults with established haemophilic arthropathies were evaluated twice using 3DGA over a time period of 18 weeks [15]. Unexpectedly, the between-period comparison revealed a tendency towards modifying the segmental joint function, but most importantly an overall infraclinical deterioration of gait pattern, characterized by a minor deterioration of the pendulum-like mechanism of gait, which is indicative of the subject's ability to save energy while walking. This study gives us some indication about the capacity of 3DGA responsiveness, that is, sensitivity to change, when used in cohort studies.

The reproducibility of 3DGA was also assessed by comparing the gait assessment of the same evaluator over time [15]. Testing the reproducibility of 3DGA and estimating the change required to exceed the measurement error are necessary in order to ensure that the error involved in the measurement is small enough to allow detecting actual changes in the patient's gait. Eighteen patients with haemophilia were tested by comparing gait variables during two sessions performed by the same investigator at baseline and after a mean followup of 18 ± 5 weeks (range 13–33). At the time of evaluation, patients had been free of acute joint or muscle bleeding for the last 30 days. Gait analysis was sufficiently reproducible regarding spatiotemporal parameters (step length and frequency) as well as kinetic, mechanical, and energetic gait variables. The kinematic variables were reproducible in both the sagittal and frontal planes [15].

### 3.2. Gait in Patients with Haemophilia with Multiple Joint Impairments

Few studies have assessed the changes produced by multiple joint impairments (MJI) of the lower limbs on gait. In a previous paper, we investigate the kinematics, cost, mechanical work, and efficiency of walking in 31 haemophilia patients with MJI, with the results being compared with healthy subjects walking at the same speed [16].

In PWH with MJI, an overall increase elevation of the vertical displacement of the CoM was observed. This should have normally resulted in increased mechanical energy being produced as more mechanical energy was needed to redirect the CoM vertically. Surprisingly, however, the mechanical work was not found to increase, which may be explained by the strategy of PWH to conserve part of the muscle mechanical work and save mechanical energy via an efficient pendulum exchange between potential and kinetic energy, that is, efficient recovery. In PWH with isolated ankle arthropathy, the recovery attains up to 70% in comparison with 60%–65% in healthy subjects. Such an improved recovery is quite unusual, being only reported in African women carrying heavy head-supported loads [17]. In PWH, the pendulum mechanism may therefore be a compensatory mechanism, a strategy to help maintaining a reasonable amount of the muscle mechanical work and saving energy despite significant joint impairments.

### 3.3. Importance of Preserved Joint Range of Motion in the Metabolic Economy of Gait

As you are done reading half of this paper, you decide to take a stroll while carefully holding a full cup of boiling coffee, and here each step the coffee bobs up and spills out of your cup. Unconsciously, you flex your legs to walk smoothly, but why does this bobbing occur when you have the ability to walk flat? In fact, extreme flat walking costs more energy than normal walking [18, 19]. The more economical mode of walking is an intermediate strategy in CoM displacement between extreme flatness and bouncy walking, that is, a CoM sinusoidal pathway of low amplitude (3-4 cm) (Figure 2). 

This intermediate strategy in CoM displacement associated with relatively straight-legged walking is achieved by peculiar movements in the lower limb joints that enable our legs to behave neither as stiff struts nor as compliant ones [18–20]. These peculiar limb joint movements are called “gait determinants” because they were considered paramount in human bipedalism, as they enable a smooth progression of the body through small fluctuations of the CoM displacement to conserve energy. For instance, if we were to walk with the legs as rigid sticks without the foot, ankle, or knee mechanisms but with the equivalent of a hip joint that permits flexion and extension only, the body would have to be elevated approximately 10 cm, which is double the normal vertical displacement usually seen in normal walking. These abrupt changes in the direction of motion would require a high expenditure of energy [20].

**Figure 3:**
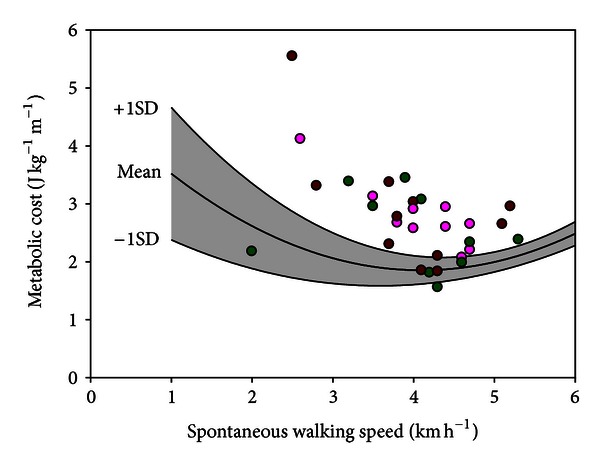
Net metabolic cost in 31 patients with haemophilia as a function of spontaneous walking speed. The severity of joint impairment is arbitrarily classified as “mild” (green symbol, uni/bilateral ankle arthropathy, *n* = 10), “moderate” (pink symbol, uni/bilateral ankle arthropathy + unilateral knee arthropathy/total knee replacement, *n* = 10), and “severe” (brown symbol, at least bilateral knee arthropathy/total knee replacement, *n* = 11).

**Figure 4:**
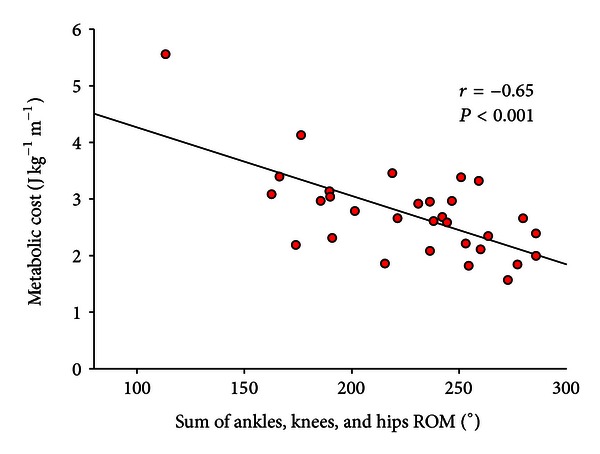
Net metabolic cost in 31 patients with haemophilia as a function of the total ROM at ankles, knees, and hips levels. The dynamic ROM of the hip, knee, and ankle was calculated as follows: ankle: A3-A2 (A3-maximum plantar flexion at pushoff; A2-maximum dorsiflexion in stance); knee: K4-K3 (K4-maximum flexion in swing; K3-maximum extension at preswing); hip: H2-H1 (H2-maximum extension in stance; H1-flexion at initial contact). The ROM of lower limb joints were calculated for both sides and then summed. *r* = Pearson product moment correlation.

Previous studies showed that one major “gait determinants” are foot movement [21]. In PWH, with the loss of some of the major determinants as a result of MJI, the strategy of vertical CoM displacement reduction is compromised leading indirectly to increase in metabolic expenditure, that is, increase of metabolic cost (Figure 3). This theory is confirmed as metabolic cost was dramatically increased in PWH and highly correlated to a loss in joint ROM at ankles, knees, and hips level (Figure 4) [16]. For instance, in PWH with isolated ankle arthropathy, the increase in metabolic cost was proportional to ankle dysfunction, that is, the less the ankle power was generated, the more the metabolic energy was consumed and more efficiency of walking was impaired [22]. The disruption to the normal walking process by an orthopedic disorder in PWH thus appears to generate mechanical and metabolic changes that follow a continuum linked to the progressive loss of mobility into the joints (Figure 4).

### 3.4. How to Explain the Increase of Metabolic Cost in PWH?

An important contributing factor may be the energy related to balance control. It is possible that PWH with joint arthropathy present an impaired balance control capacity. Compensations in order to increase the dynamic stability may elicit a substantial and meaningful metabolic energy demand. Stiffening the body through co-contractions could be one of these strategies. As these cocontractions from antagonist muscles do not produce any movement, they consume a lot of metabolic energy with no apparent work produced. Part of the muscular work may also be used to overcome internal friction and viscosity in the ligaments, muscles, or joints in order to deform the body segments. This work is supposed to be greater in PWH as joint stiffness and muscle fibrosis are common complications of arthropathy [23].

### 3.5. 3DGA Tested as an Assessment Tool in the Context of a Clinical Study

In light of these methodological and fundamental investigations, 3DGA appears to be a powerful tool to quantitatively characterize the locomotor functions of patients with gait disturbances, including PWH. 3DGA can also be used in clinical trials to objectively quantify the effects of a conservative orthopedic treatment in PWH.

Foot deformities are common in patients with haemophilic ankle arthropathy and are often responsible for discomfort when patients walk or stand for long periods. Currently, there are no validated conservative orthopedic options for managing haemophilic ankle arthropathy. No study has yet addressed the potential benefits and practicalities of foot orthoses in ankle haemophilic arthropathy. As relatively cheap (in comparison with factor replacement) conservative treatment, foot orthoses will likely make a substantial difference in terms of comfort and function for patients with limited access to replacement therapy.

We experimentally investigated the effects of custom-made orthopedic insoles and shoes in PWH with ankle arthropathy, with special attention given to pain and gait [24]. We suggested that orthoses may have beneficial effects, as they provide significant pain relief and comfort improvement, with minimal side effects. More specifically, insoles had limited impact on gait pattern, whereas orthopedic shoes significantly improved the propulsive function of the ankle (increased ankle power). Increases in ankle moment and knee flexion in the stance phase also suggested that patients with orthopedic shoes experienced improved weight acceptance, probably due to improved comfort and reduced ankle pain. Biomechanical changes induced by these orthoses were, however, insufficient to influence knee and hip kinematics and kinetics in addition to mechanical and energetic variables [24].

### 3.6. Directions for Future Research: Emerging Clinical Assessment Tools in the Paediatric Population with Haemophilia

Recent work evaluating joint status and functional impairments associated with lower limb haemophilic arthropathy using specialised laboratory equipment to study biomarkers of human motor performance has shown that when young preadolescent boys with haemophilia with a history of ankle joint bleeding are compared to age and size matched typically developing peers, lower limb muscle strength and size are reduced in hemophilic boys [25]. Alterations in kinematic and kinetic gait patterns [26, 27] have also been observed. Muscle strength and atrophy appear to underlie these biomechanical changes [25, 28].

With respect to the pediatric population, preliminary data from our group reported that in clinically asymptomatic children with a previous history of joint bleeds, kinematic and kinetic responses to perturbations may be observed, principally at the ankle level. To further complement this research work, we intend to conduct an ambitious multicentric study to better understand the biomechanical consequences and adaptations of ankle arthropathy in children. This project aims to explore several aspects of ankle function (viscoelastic properties of muscles as well as motion of the small joints of the foot) that have never been explored before. We also intend to compare this functional assessment with a thorough radiological evaluation by magnetic resonance imaging and ultrasound.

## 4. Conclusions

Musculoskeletal impairments in PWH may stem from structural and functional abnormalities, which have traditionally been evaluated radiologically or clinically. However, these examinations are performed in a non-weight-bearing supine position. Our studies showed that three-dimensional gait analysis can be an innovative tool to exclusively focus on the functional component of the joints during walking. This is of the utmost importance, as pain induced by weight-bearing activities significantly influences the functional performance of the arthropathic joints. Our studies showed that in PWH, the more the joint function was altered, the more the metabolic energy was consumed. 3DGA analysis could highlight the effect of an orthopedic disorder in PWH during walking which can be a useful tool for clinicians. Integrating biomechanical research with clinical research has the potential to improve our monitoring of the early blood-induced changes in the physical status of the hemophilic joint.
